# Why perfect policy coherence is unattainable (and may be ill-advised)

**DOI:** 10.1007/s11077-025-09582-9

**Published:** 2025-08-05

**Authors:** Paul Cairney

**Affiliations:** Division of History, Heritage and Politics, University of Stirling, Stirling, UK

**Keywords:** Policy coherence, Policymaking integration, Policy mainstreaming, Policy mix, Implementation gap, Coherence gap, Top-down and bottom-up perspectives

## Abstract

Classic studies of ‘perfect’ policymaking use an ideal-type to identify and reflect on policymaking in the real world. I use this approach to review studies that seek policy coherence, show how policy theory insights help to identify real-world dynamics, and prompt debate on what would constitute perfection. The ideal-type ‘perfect policy coherence’ initially helps to identify barriers to policymaking integration and the production and delivery of a coherent policy mix. It has the following elements. There is high and consistent attention to a problem, and solving that problem is the highest strategic priority. There are effective means to produce evidence-informed policy and manage competing beliefs and interests. There is a perfect means to coordinate policy implementation. The strategy works as intended. There are clear and agreed measures of success, and meeting targets signals substantive progress. The policy strategy and mix are credible and durable. I use the ideal-type to show that fragmentation and incoherence are inevitable in the real world. I argue that this ‘coherence gap’ - between ideal-type and real-world policymaking - is not all bad, then compare competing - ‘top down’ and ‘bottom up’ - ways to seek integration and coherence.

## Introduction

This article envisages ‘perfect policy coherence’ to explain a ‘coherence gap’ and prompt debate on how to close it. The coherence gap is between an *ideal-type*, used to identify abstract elements of key aims, and *real-world policymaking*, used to demonstrate inevitable limits to perfection. The word ‘perfect’ is provocative, to prompt debate on what it would look like and how far we should go to achieve it. This approach is central to policy process research, which uses ideal-types to compare an artificial construct (it could not exist in the real world) with policymaking in the real world, and reflect on how to respond. Perfect rationality is a device to explore how policy actors deal with bounded rationality (Simon, 1976). ‘Perfect administration’ helps to identify the ‘limits to administration’ such as the limited control of one coordinative centre (Hood, 1976: 17–27). ‘Perfect implementation’ explains an ‘implementation gap’, such as when a policy lacks clarity, compliance, and resources to deliver (Hogwood and Gunn’s, 1984: 198 phrase “why ‘perfect implementation’ is unattainable” is the initial inspiration for this article).

Therefore, these *ideal-types* serve two functions: to make systematic comparisons with policymaking reality and prompt debate on policymaking *ideals*. They prompt us to ask: from whose perspective would this model represent perfection? For example, ‘perfect implementation’ describes a ‘top-down’ perspective that describes perfection from the perspective of central government policymakers. It was challenged by ‘bottom-up’ scholars focused on the perspective of organisations and individuals charged with policy delivery (Barrett & Fudge, 1981). They argued that top-down perspectives provided flawed explanations for complex organisational activity *and* undervalued the legitimate role of actors making and delivering policy outside of the ‘top’ of government. Although the implementation debate petered out, debates on the premise of perfect administration and implementation live on in new forms. For example, contemporary discussions of governance suggest that the pursuit of highly centralised control is not realistic, and may not be the most effective or democratic solution, in a multi-centric policy process (Hooghe & Marks, 2003; Cairney et al., 2019). Hence, provocative visions for ‘perfect’ policy processes remain essential, to identify empirical reality and inform normative debate on how to respond.

Although this conceptual approach is well known within the policy sciences, it has untapped value for scholars from many other disciplines, taking an interest in policy and policymaking, but less aware of the counterintuitive dynamics of policy processes or lessons from historic debates. This value is high in relation to an ideal-type approach to the dynamics of *policymaking integration in the service of policy coherence*. Most journal articles are by scholars outside of political science or the policy sciences, seeking to promote or understand government initiatives to address problems such as climate change and global health inequalities, and often frustrated with a lack of integration and coherence (see reviews by Tosun & Lang, 2017; Trein et al., 2023; Aoki et al., 2024a). In that context, both aims seem like valence issues: who would want policymaking *fragmentation* or policy *incoherence* when dealing with urgent existential policy problems? Yet, this reference to the self-evident value of coherence downplays or ignores the politics and complexity of policy change (Yunita et al., 2022).

Such interdisciplinary research would benefit from the specialist policy sciences insights that are familiar to policy process scholars, such as to show that fragmentation makes sense and incoherent policy is almost baked into complex and multi-level policymaking systems (Cejudo & Trein, 2023; Howlett, 2014; Cairney, 2025a; Cairney et al., 2022). This small subset of a large ‘policy integration’ literature shows that there are practical benefits to maintaining silos (to develop specialist expertise) or policy subsystems (to maintain trust between a manageable number of actors), and normative benefits to distributing policymaking responsibilities across many elected and unelected venues (some have electoral legitimacy, some have legitimacy through professionalism and independence). In that real-world context, actors in each venue have good reasons to protect their established ways of thinking and working, so they exercise power to frame issues in their favour, maintain distinctive rules and policies, and produce their own policy instruments that contribute to an overall mix. Further, the resources of a single ‘centre’ to coordinate policy across these venues is limited, and the incentive to use them varies.

Even with that logic alone, we would reject the idea that the pursuit of coherence could be a technical exercise in central planning to produce one optimal outcome, or that one integrated strategy could win the day. Rather, policy theories show that there is inevitable– and often valuable - political contestation to determine which levels or types of government have a legitimate role in making and delivering policy. Hence, from a purely technocratic perspective, we see damaging fragmentation and incoherence to be solved. From a wider political perspective, we see the pursuit of policymaking autonomy to tailor policy to each context. In that context, it is simple to describe the policy and policymaking problem, but not to produce a clear and well-supported solution. Therefore, if actors are frustrated with a lack of progress, ill-equipped to understand the politics and complexity of policymaking, and seek simple centralised responses, they may contribute to the very problem they bemoan.

To demonstrate, I construct the ideal-type ‘perfect policy coherence’ and relate it to a wider examination of empirical and normative insights from policy process research. My approach differs somewhat from Hood (1976) and Hogwood and Gunn (1984), who construct ‘perfection’ largely from first principles to compare a simple model with complex reality. Here, I base this construction on my systematic review of the 705 texts listed by two previous systematic reviews: Trein et al. (2023; Trein, 2022) examine the conceptual, methodological, and theoretical sophistication of empirical ‘policy integration’ (PI) studies; while, Aoiki et al. (2024a; 2024b) explore the sectoral and geographical application of ‘whole-of-government’ (WG) approaches (hereafter, I use the PI abbreviation to include WG) (see Cairney, 2025a; 2025b for extensive background information on the wider literature). My aim is to demonstrate the unresolved dilemmas that emerge when we consider the differences in approaches or expectations in the overall field of study. The value of this new review is to show that, if we add up all the requirements from coherence advocates, they combine to produce (1) a long and unrealistic account akin to an ideal-type, *and* (2) a vision that contains unresolved tensions between relatively top-down and bottom-up conceptions of integration and coherence. What begins as a valence issue (who would not want coherence? ) soon becomes a thorny contested issue (what exactly is it, and what should we do to secure it? ).

Setting up this ideal-type allows us to examine a simple comparison between each requirement and reality, relate it to theoretical and empirical studies of integration, and prompt debate on whose vision of coherence to seek. To that end, I draw on illustrative examples from the field, and more general policy theory-informed insights, to explain why this ideal-type is unattainable. Each empirical example highlights a tendency to identify a requirement but find a more disappointing result. The wider theoretical literature shows that non-integration and incoherence are features to adapt to within policymaking systems, not bugs that could realistically be designed out (Cejudo & Trein, 2023). Hence, empirical studies will inevitably identify a ‘coherence gap’ between the stated intentions of reformers and the outcomes, but not necessarily know how to respond.

In that context, I identify competing visions for policymaking integration and whole-of-government approaches in the service of policy coherence. Some seek to combine elements of perfect policy coherence to encourage and monitor progress. However, my analysis of such models suggests that many governments and researchers have different ideas of the meaning and main purpose of policymaking integration, such as to: prioritise centralised top-down direction or decentralised and bottom-up collaboration, see coherence from the perspective of one sector at the expense of others, or ensure that one priority sets the integration agenda for the rest. Consequently, the sum of indicators of coherence is an incoherent list, reflecting unresolved differences in aims.

Overall, I show that no single ideal-type of perfect policy coherence can present a clear, consistent, and universally supported normative position. There is no simple ideal to inform targets and measures of progress. Rather, the inconsistency of the literature reflects different expectations for democratic and effective government as well as inevitable gaps between ideal-type and policymaking reality.

## What is perfect policy coherence? Comparing an ideal-type with reality

Policy coherence is a rather vague term, described initially as: ‘various policies go together because they share a set of ideas or objectives’ (May et al., 2006: 382). It is analytically distinct from policymaking integration, at least if policy coherence describes the aim and policymaking integration the process to achieve it (Cairney, 2025a; although ‘policy integration’ is the more established term for both elements). If we combine both elements, the broad theory of change of aspirational PI research is that more coordinated policymaking will produce more effective policy, such as to reduce contradictory practices and boost the mutually-reinforcing benefits of multiple policies (Pollitt, 2003: 35; Tosun and Lang, 2017; Aoki et al., 2024a). Hence, key definitions tend to emphasise the process to support the policy, such as Tosun and Lang’s (2017: 553) definition of PI as (1) ‘the collaboration of actors from two or more policy domains’ to (2) ‘integrate aims and concerns derived from one policy domain into another’. Similarly, the process for ‘integrated policy design’ helps to improve the policy mix:

“Integration is the replacement of specific elements of existing policy ‘mixes’ or ‘regimes’– the goals, objectives and calibrations of existing policy tools and goals– by a new policy mix, in the expectation of avoiding the counterproductive or sub-optimal policy outcomes that arise from treating interrelated policy regimes and components in isolation from one another” (Rayner & Howlett, 2009: 99–100).

This combination of aims for process and policy helps to explain why so many requirements arise from studies of policy coherence: *they describe not only where to go*,* but also the many steps to get there*. The result of combining their requirements is a long shopping list consisting of processes to produce coherent strategies, coordinate strategies across levels and types of government, seek participation and consensus among actors, connect strategy to a policy mix, deliver policy, evaluate, and ensure that policy is fit for purpose for the long-term.

I synthesise these insights to construct the indicators of perfect policy coherence in Table 1. Then, I summarise each requirement and use illustrative examples to compare requirement with reality (see also Sahin & Feaver, 2013: 1075; Scobie, 2016). This approach follows Hogwood and Gunn’s (1984: 198–206) exposition of ‘perfect implementation’. Indeed, many requirements have a similar feel, such as to seek clear and well communicated aims, technical feasibility (it will work as intended if implemented), assign sufficient resources to delivery, achieve administrative compliance and minimal opposition from groups, and avoid the unintended consequences of external conditions or events. Further, perfect policy coherence often resemblances Hogwood and Gunn’s (1984) wider exposition of a policy cycle coordinated from the top, to ensure that: there is an orderly policy process, all relevant actors know how to contribute to each stage, a coherent strategy translates into a coherent policy mix with resources behind implementation, and there is evidence that policy works successfully when implemented.

**Table 1:** Indicators of perfect policy coherence

Category	Example
Procedures	The policy process is clear, consistent, and predictable
Agendas	There is high attention and priority to a policy problem The problem’s definition (framing) is clear and consistent
Analysis	Problem diagnosis is evidence-based Solutions will work as intended if implemented Normative beliefs about what to do align with empirical expectations
Actors	There is substantive involvement among all relevant actors Key actors share responsibility for the problem and ownership of solutions
Consensus seeking	There are meaningful and effective ways to manage competing beliefs and interests, to seek consensus through collaboration rather than imposition
Levels of government	There is a means to coordinate a coherent strategy or ensure that separate strategies do not undermine each other
Instruments	A strategy’s policy mix contains mutually supportive instruments
Implementation	Delivery resources are in place. Delivery does not undermine strategy
Outcomes	The strategy works as intended when implemented The policy mix sends clear signals and recipients experience coherence
Monitoring and evaluation	There are agreed targets. Reaching a target signals substantive progress. There is high agreement on indicators and measures of success
Fit for purpose (over time)	The strategy, mix, and delivery are credible in the short-term and durable over the long-term

Source: author’s own ideal-type construction, synthesised from 50 + articles in Cairney (2025b)^1^. Each article contributes to at least one Table 1 element, but responsibility remains with the author

^1^ E.g. Benson & Lorenzoni, 2017: 1924; Lafferty & Hovden, 2003; Candel & Biesbroek, 2016; Biesbroek and Candel, 2020: 64; Deters, 2018; Delany-Crowe et al., 2019; Clar, 2019; Daniell et al., 2011; Danaeefard et al., 2019; Daneshpour et al., 2018: 259; Domorenok, 2019; de Jong & Pacheco, 2016; Endl, 2017; England et al., 2018; Falaleeva et al., 2011; Farmery et al., 2019; 2020; Feindt, 2010; Fertel et al., 2013; Feola et al., 2019; Velázquez Gomar, 2014, 2016; Hanssen et al., 2016; Hardy & Koontz, 2010; Huttunen, 2015; Jaeger & Michaelowa, 2015; Leal Filho et al., 2016; Le Blanc, 2015; Lowe et al., 2018; Lyles et al., 2014; Lyytimäki, 2011; Mayor et al., 2015; Metz & Glaus, 2019: 7–8; Morf et al., 2019: 206; Morin & Orsini, 2014; Mu and de Jong, 2016: 62; Nilsson et al., 2012, 2018; Nunan et al., 2012; Niedertscheider et al., 2018: 11; O’Halloran, 2021; Pham-Truffert et al., 2020; Pilli-Sihvola & Väätäinen-Chimpuku, 2016: 465; Reyes-Mendy, 2014; Rietig, 2013; Rietig & Perkins, 2018; Simoes et al., 2015: 443; Sixt et al., 2020: 13–14; Smith et al., 2014: 2625; Söderberg, 2011; Stroß, 2017; Storbjörk & Isaksson, 2014; von Lüpke & Well, 2020; Waylen et al., 2019; Willems et al., 2021: 86

### Procedures

*Requirement*. The means to make policy are consistent, understood, and predictable. Policy actors know what is decided and when, and how they can provide evidence or foster collaboration and learning at each stage. Learning processes can include sharing empirical information, trial-and-error, and successful argumentation to shape the beliefs of other actors.

*Reality*. The absence of a well-understood process to anticipate or resolve multiple sources of incoherence, including: unresolved competing aims among many actors, competing central-local priorities, competing sectoral aims, and/or transnational issues when national outcomes do not fulfil international commitments (e.g. Mallory, 2016 on fishing policies in China).

### Agendas

*Requirement*. There is high attention to a well-defined problem. There is an agreed, consistent framing of the problem, not a messy compromise of beliefs. The problem is high priority to the extent that a commitment to policy coherence is not undermined by other aims or co-opted for other purposes.

*Reality*. Incoherence reflects insufficient policymaker attention to, or concern for, issues raised by low-power actors (e.g. Feola et al., 2019; Soria et al., 2020; Füller et al., 2018 on land grabbing and corruption, ‘urban sprawl’ at the expense of ‘ecosystem services’, and the use of public space at night). The aim is coherent but relatively low priority, such as when there is weak support for policies to boost population health and health equity, and these policies are undermined by trade agreements that boost the production and consumption of ‘unhealthy commodities’ or reduce equitable access to healthcare (Ruckert et al., 2017: 86).

### Analysis

*Requirement*. There is high alignment of stated goals, aims, and objectives to address the problem. Normative beliefs (this is what we should do) align with empirical or causal beliefs (this is what we expect to happen if we do it). This alignment is underpinned by comprehensive technical analysis. The diagnosis of the problem, and identification of solutions, is based on high quality evidence and analysis. This requirement is akin to technical feasibility: a policy will work as intended if implemented. Feasibility is facilitated by a full understanding of the policy context, consistent aims, explicit and substantive engagement with inevitable trade-offs between aims, and proportionality in relation to the size and scale of the problem. There is a clear theory of change that connects means to ends, such as to determine how each instrument contributes to an overall objective. If many instruments involve different target groups or mechanisms of delivery, there is a means to join-up thinking.

*Reality*. The most frequent issue with government agendas is the superficial use of PI language without evidence of substantive analysis, a clear theory of change, or a means to identify and address trade-offs (Cairney, 2025a, b). Consequently, there are unresolved and often high-conflict trade-offs between aims (e.g. the impact of renewable energies on biodiversity - Rietig, 2013: 298). Belief conflict can combine ‘normative beliefs regarding policy objectives’ and ‘causal beliefs’ about the intended and likely impact of policy instruments, and unresolved tensions perpetuate high-level policy incoherence (Skovgaard, 2018: 350; Selianko & Lenschow, 2015; Schunz et al., 2021).

### Actors

*Requirement*. There is substantive involvement by key governmental and nongovernmental actors in multiple policy sectors, rather than policy being contained in one or few subsystems. These actors take individual responsibility *and* foster a binding sense of collective responsibility, to produce widespread ownership of a coherent approach.

*Reality*. The varying involvement of actors is a routine feature of policy communities or subsystems, to the extent that: ‘policy integration is in permanent political tension with the sectoral logic of policymaking’ (Cejudo & Trein, 2023: 9; 11; see also Jordan & Maloney, 1997). This separation may also be apparent within broad networks, such as when climate change networks do not fully connect mitigation and adaptation efforts or include enough public and private sector actors essential to implementation (Locatelli et al., 2020).

### Consensus seeking

*Requirement*. There is an effective means to manage the competing interests whose demands could contribute to incoherent messy compromise. Stakeholder engagement goes beyond seeking policy relevant information and communicating intent, towards relationship and trust building with participants. Policy is done with you, not to you. This approach is driven by the knowledge that too much imposition now (to try to resolve conflict) stores up or displaces conflict for later, especially if multiple aims can be described as coherent in the vague abstract but in conflict in practice. Process design, to include actors in networks, is as important as institutional design.

*Reality*. Poorly designed and superficial stakeholder engagement is pervasive (Vasileiadou & Tuinstra, 2013), undermining essential collaboration to produce a shared understanding of the problem and agree solutions (Zegras & Rayle, 2012: 313). Unless properly resourced, a well-designed policy and collaboration strategy does not produce sufficient collaboration in practice (Satumanatpan & Chuenpagdee, 2015: 16). Conflicts of beliefs among advocacy coalitions lead to policy changes without the participation of some groups, producing low ownership and long-term sustainability (Sarvašová et al., 2013: S70-71). Policy design may enjoy high legal integration that is not matched by ‘actor integration’, ‘to the extent that actors disrespect integration mandated and incentivized by laws’ (Metz et al., 2020: 8). Or, in some contexts, actors see pragmatic value in ‘productive bricolage’ to manage rather than remove goal conflicts among actors (van Oosten et al., 2018: 69).

### Levels of government

*Requirement*. Multiple authoritative organisations, spread across each level of government, coordinate a single coherent strategy. Or, their separate strategies complement each other. For example, international commitments remain feasible in relation to national strategies, and there is clear vertical integration between international, national, and subnational strategies.

*Reality*. High level agreements tend to be vague strategies designed to avoid opposition or conflict among actors with competing interests, with little capacity to follow through (Cejudo & Trein, 2023: 15–16; Russel et al., 2020: 15; Deters, 2018: 359; Sotirov et al., 2020: 19). An under-developed high-level strategy reflects a lack of senior policymaker attention and support, unresolved sectoral or organisational competition, and limited staff resources to make sense of key aims in local implementation (Russel et al., 2018: 48–49 on EU climate and marine policy). For example, a lack of integration between elements of the UN Sustainable Development Goals (SDGs), and between SDGs and national strategies, is routine (Koide & Akenji, 2017; McGowan et al., 2019: 43). Such disconnects are multiplied when many policymaking organisations– spread within or across policy sectors and levels of government - have some powers to produce some instruments for different purposes and make or deliver policy at different times (Cairney et al., 2022; Bahn-Walkowiak & Wilts, 2017: 165; Candel & Biesbroek, 2016; Biesbroek & Candel, 2020: 64; Howlett et al., 2017: 72; Righettini & Lizzi, 2020; Sandström et al., 2020; Simoes et al., 2015). Each authority can go its own way, creating tensions when one venue bemoans its lack of powers and the dilution of policy by other governments (e.g. Cairney et al., 2021: 426-7 on multi-level gender mainstreaming).

### Instruments

*Requirement*. There is a well-defined policy mix containing mutually supportive policy instruments at a strategic level. Policy instruments from some sectors do not have direct or indirect negative consequences on others. The balance between state, market, and individual responsibility is clear, and actors are aware of their incentives or responsibilities to act, and any punishments for non-compliance.

*Reality*. Multiple studies describe the coherent policy mixes that *could* be adopted, but this is not the same as actual adoption. Examples include: Mwangi et al. (2021: 11) on ‘priority interventions’ for non-communicable diseases, Vimpani (2005: 116 − 20) on ‘upstream’ interventions to prevent ‘substance misuse’, Brendehaug et al. (2017: 1261) on sustainable tourism, Downs et al. (2015: 478) on measures to reduce trans-fat consumption, Lowitt et al. (2016) on ‘food security’ coherence, and van Stigt et al. (2013: 226) on sustainable EU cities.

Policy mixes *could* also be supported by a well-defined range of ‘coordination instruments’ to support alignment with an integration strategy, divided into *management* (strategic, financial, information sharing, and review mechanisms) and *structural* (including reorganisations and mergers, internal markets, partnerships, and coordinating bodies) (Verhoest et al., 2007: 345; see also Antwi-Agyei et al., 2017: 5; Aubrechtová et al., 2020: 4; Becken et al., 2020: 1611; Baulenas & Sotirov, 2020: 3; Benson & Lorenzoni, 2017: 1924; Kelleher et al., 2019; Koff, 2020: 414; Oliveira et al., 2019; Okpara et al., 2018; Reyes-Mendy, 2014). However, very few studies identify a government’s use (or even description) of a substantive and coherent policy mix (Cairney, 2025a). When they do, they find that some instruments tend to be more developed than others, including: greater substantive action for ‘forest bioenergy’ than ‘carbon sequestration’ to sustain ‘forest ecosystems’ (Makkonen et al., 2015: 161); and, more progress on EU spatial planning in ‘environmental, transport, cultural heritage, energy and waste policies’ than ‘sectors such as health, education, information and communication technology-digitalization, and retail policy’ (Nadin et al., 2021: 796). Or, there is varying success in the adoption of ‘procedural policy instruments’ to foster cross-government collaboration and network governance, such as when Lang (2019: 278) describes greater UK than German progress to manage three subsystems essential to reducing the costs of health technology.

### Implementation

*Requirement*. Strategic alignment and coordination translate to implementation. Coherent policy mixes are well-resourced and there are no legal loopholes or impediments to delivery.

*Reality*. Multiple studies describe the ‘barriers’ to implementation when seeking to go beyond coherent strategies (Karlsson-Vinkhuyzen et al., 2018: 134; Zinngrebe, 2018: 157; Miller et al., 2018; Waylen et al., 2019). Often a major strategy is too (a) vague, superficial, or tokenistic, and (b) disconnected from implementation or delivery, to give any confidence on its likely effect. Governments produce warm rhetoric that describes integrating everything and therefore nothing, or produce a coherent strategy unaccompanied by a clear sense of who would deliver with what resources (e.g. Rouillard et al., 2013: 384; Isoaho et al., 2019; Kalaba et al., 2014; Larsen & Powell, 2013; Ranabhat et al., 2018: 975; Steurer & Berger, 2011; Storbjörk & Isaksson, 2014: 1039). Or, there is an implementation gap despite more substantive legislative action. For example, Rudd et al. (2018: 4) find high legislative coverage for ocean ecosystem-based management, but subject to ‘conflicting interpretations’ and limited incentives and resources to cooperate within and across countries, in a wider context where stakeholder and citizen engagement is often limited, and implementation is vulnerable to political whims and the difficulty of making a business case for this work (2018: 5–15).

### Outcomes

*Requirement*. Implementation contributes to coherent policy outputs or outcomes. A strategy works as intended when implemented. A collection of instruments with different target populations still sends clear and consistent signals to practitioners and recipients. Policy recipients experience coherence when making sense of multiple instruments or signals from multiple authorities.

*Reality*. Even well-regarded policy instruments do not work as intended when implemented. For example, Environmental Impact Assessment (EIA) does not ‘reduce disaster risks of development projects’ (Hapuarachchi et al., 2016), and an ‘almost exponential increase’ in ‘flood risk management policies’ has not reduced flood risks and damage (Metz & Glaus, 2019: 1).

### Monitoring and evaluation (Policy success)

*Requirement*. There is high agreement on well-defined milestones and targets. Meeting a target is a clear sign of substantive progress and a win for policymakers and stakeholders. There is high agreement on the measures and means to evaluate progress and success.

*Reality*. There is no commonly agreed language to describe, or metric to measure, coherence and long-term progress (May et al., 2006: 383). Policy evaluation is notoriously contested, and actors may use three different measures of success: it boosted government popularity (‘political’); it gained legitimacy and maintained support (‘process’); and, outcomes matched stated goals (‘programmatic’) (McConnell, 2010; see also Scott & Boyd, 2023). Consequently, sophisticated evaluations of programmatic success are rare (Trein et al., 2023: 9–10; Jordan & Lenschow, 2010: 155; Candel, 2017; Careja, 2011) and struggle to explain good progress (Klement & Blokland, 2023: 1205).

Instead, studies tend to identify process-based proxies of success. Simeonova and van der Valk’s (2010: 1424-25; 2016) nine ‘success factors’ for local environmental PI planning include meaningfully decentralised authority and stakeholder and citizen awareness or buy-in. Simeonova et al’s (2019: 126 − 30) success factors for integrated ‘ecological and urban development’ include a clear sense of’ shared responsibilities’. Indig et al’s (2019: 23) questionnaire measures the extent to which participants: understand the aim of an obesity prevention partnership, see the value in contributing their time, and agree that ‘partners are achieving more together than they could alone’. Blackburn’s (2016: 361-3; 364-5) measures of success of ‘one-stop shopping for government services’ include citizen satisfaction with services and employee morale.

### Fit for purpose (over time)

*Requirement*. The strategy, mix, and delivery are credible in the short-term and durable over the long-term. Here, time is a flexible proxy for longer term changes that should not undermine policy coherence. For example, the problem framing or policy response changes in proportion to a changing policy problem. Or, any gap between local innovation, learning, and convergence is closed quickly to ensure wider territorial coherence. Time is also used to compare the relative coherence of short- and long-term plans to deal with crises: a response should be timely and should not drift from its original purpose.

*Reality*. There is *some* cause for optimism in this category. Studies combine multiple indicators of an integration strategy’s credibility, relating to factors including the sincerity of its architects, and strategy’s inclusiveness of stakeholders and ability to broker meaningful agreement (perhaps as part of a broader focus on good governance), adaptability to changing circumstances, substance, follow-through, and connection to accountability mechanisms (e.g. Nadin et al., 2021: 796-8 on ‘integrated, adaptive, and participatory spatial planning’). Further, some positive reports of credibility describe the sense that we have been here before: there is a legacy of effective collaboration, policy change, and demonstrable success on which to build (Söderberg, 2011: 532; Gössling, 2013: 204; Plowden, 2020: 160; Wamsler & Pauleit, 2016: 82).

### From the ideal-type to ideal visions for policy coherence

This mild optimism may enthuse advocates of coherence who seek meaningful policy change. The next step may be to use multiple elements of Table 1 to establish an ideal to which to aspire (Ishii & Langhelle, 2011) then measure progress in relation to one or more elements (e.g. Guerrero and Castañeda’s, 2021 ‘policy coherence index’). Examples include: *Agendas*, to welcome more sustainability language in spatial plans (Rega & Bonifazi, 2014: 1355); *Analysis*, to better connect strategies on climate mitigation and energy security (Strambo et al., 2015); and *Instruments*, to ‘plug gaps’ in strategy when a new initiative emerges (e.g. Voyer et al., 2020 on the ‘blue economy’). More generally, studies identify ways to get beyond coherence in ‘policy discourse and negotiation’ towards ‘policy goals and instrument formulation’ and implementation (von Lüpke & Well, 2020: 841-2). This progress requires good evidence on how progress in each sector affects the others. For example, Pham-Truffert et al. (2020: 1247) draw on Nilsson et al’s (2018) ‘seven types of interactions between SDG targets’ to identify which targets create ‘co-benefits without much risk of producing trade-offs’ or benefit from progress in other areas, which targets multiply trade-offs, and which actions have the most potential to shift from negative to positive cycles.

Further, many approaches seek to combine Table 1 elements to produce a comprehensive model to measure and/or encourage coherence. For example, Niedertscheider et al. (2018: 11) draw on Mickwitz et al. (2009) to identify 9 criteria for climate change mitigation coherence: ‘*inclusion’* of all relevant issues, ‘*consistency’* of mitigation and adaptation aims, a high ‘*weighting’* of climate aims in relation to other sectors, clear ‘*reporting’* processes, sufficient ‘*resources’*, ‘*reflexivity’* or continuous learning on progress, dedicated ‘*commitment’* and *‘sanctions’* for non-compliance, key ‘*uncertainties’* are addressed, and the process is ‘*multi-level’* and ‘*multi-actor’* (see also Russel et al., 2018: 45; Šumrada et al., 2020). Zinngrebe (2018: 155 − 56) suggests that progress towards ‘biodiversity policy integration’ happens when: key ‘political sectors’ engage with biodiversity (‘inclusion’), modify their policy mix (‘operationalization’), produce a coherent mix in each sector (‘coherence’), assign sufficient resources (‘capacity’), and give the issue priority (‘weighting’). Kettner and Kletzan-Slamanig (2020: 144-5) seek to measure the European Union’s promising but mixed climate policy integration progress in relation to factors including: meaningful ‘political commitment’ (statements backed by strong policy instruments), a clear assessment and engagement with ‘functional overlap’ (trade-offs or spillovers between aims), a coherent policy mix, giving proper weight – ‘principled priority’ - to climate policy integration (CPI) over other aims, and a clear connection between long-term CPI aims and the short-term commitments of key actors. Further, Martin et al. (2014: e391-95) describe a rubric to score governments on their progress towards PI for obesity prevention (Australia), based on factors including:

- the production of a strategic plan with a clear account of the policy mix.
- regulations to prohibit the promotion of unhealthy food by industry (and fund social marketing to combat such messages).
- financial support for healthy food provision.
- ‘mandated guidelines’ on exercise and nutrition in schools and to restrict the availability of unhealthy food sales in health and social care.
- planning and infrastructure support for ‘active travel’ and to enjoy green space.
- funded healthy eating promotion, including support for workplace reforms.

This approach to coherence is also a feature of benchmarks and country rankings for tobacco control, which is often treated as a model of good practice – and clear progress – for other ‘noncommunicable disease’ policy agendas (e.g. Joossens et al., 2020; Cairney, 2019; Battams & Townsend, 2019).

## Whose vision of perfect coherence matters?

However, all such initiatives are incomplete unless they interrogate the question: from what or whose perspective should we understand coherence?

### Top-down and bottom-up perspectives on coherence

Table 1 bears a strong resemblance to the classic ‘top-down’ view of implementation, to see coherence from the perspective of one single centre of authority seeking to produce then deliver a strategy (Hogwood & Gunn, 1984). This approach suggests that a ‘coherence gap’ is on a grander scale than an ‘implementation gap’ since it relates to a mix of instruments and the need for each instrument to be implemented to contribute to overall coherence. Too-low attention and energy to one instrument affects the whole mix, so coherence requires continuous and comprehensive top-down activity.

However, key parts of the literature identify two related problems with this perspective and expectation. First, experiences of the most-researched countries – including the UK and Australia - suggests that central governments do not respond well to PI constraints (Carey et al., 2015a, b: 167). Their previous New Public Management reforms exacerbated coordination problems by expanding the number and autonomy of government agencies and delivery bodies, then they responded with unclear and ill-resourced joined-up-government strategies, while prioritising central control over local autonomy and wider collaboration (e.g. Campbell, 2007; Flinders, 2002; Matthews, 2013: 55–79 on the UK). There were periodic attempts to reform, which produced a limited positive effect, major unintended consequences, and demoralisation or scepticism among the local actors responsible for making sense of top-down aims at the bottom (e.g. Molenveld et al., 2020, 2021).

Second, there is more than one legitimate way to pursue coherence. For example, multiple studies explore coherence from a variety of regional and local perspectives. Here, studies find different priorities and policy instrument-use among each level of government responsible for delivering PI (Sheng, 2021). If so, policy may seem coherent in an overarching national strategy, but not among the local governments or street-level bureaucrats trying to make sense of it in practice (Sevä & Sandström, 2017, drawing on Lipsky, 1980; van Stigt et al., 2013: 231).

In such cases, researchers make the case for subnational governments or local actors to have more responsibility, resources, and autonomy to address issues that transcend jurisdictional boundaries and manifest differently in relation to context (e.g. Antonson et al., 2016; Berg et al., 2010; Velázquez Gomar, 2016). This need for flexibility is strong when policies address environmental or ecosystem issues, there is a disconnect between jurisdictional and natural geographical boundaries, and local expertise is essential (Sandström et al., 2020: 5–6). Or, this case combines empirical and normative elements, such as when Sullivan (2003: 366) argues that a supportive architecture for coherence is most visible at a local government level, as the only organisation and scale able to produce coherent delivery coupled with direct representation and citizen participation. Further, local participatory governance is only meaningful when local governments have the autonomy and resources to act on the results (Aurich-Beerheide et al., 2015: 390).

In terms of Table 1, the emphasis is more on the need to involve a full range of subnational actors from the beginning, engage in meaningful consensus-seeking or collaboration to define and address the problem, then give subnational governments the resources to collaborate with local stakeholders, rather than treat coherence as a need to close the implementation gap between top-down strategy and outcomes via obligation and punishment for non-compliance.

### Sectoral perspectives on coherence

Similar under-explored tensions exist between separate sectoral efforts towards integration (Khan et al., 2018). Without an agreed focal point for integration, different actors may perceive *their* issue to be at the heart of integrative efforts and seek to bring in others to *their* sector. If so, an issue treated as the main priority for one subsystem may be largely ignored in another. For example, PI for ‘food’ can relate to economy, development, trade, employment, climate mitigation and adaptation, waste, energy, agriculture, fishing, poverty, security, gender, obesity and/or child or adult nutrition strategies (Farmery et al., 2019; 2020; Feindt, 2010; Friel et al., 2019; Moschitz, 2018; Namugumya et al., 2020). Then, an additional frame such as food waste, access, use, or availability (or adding a governance scale such as cities), can multiply confusion about the involvement of actors and ideas in each context (Righettini & Lizzi, 2020: 130; Sibbing et al., 2021: 59; Sonnino et al., 2019).

### Critical perspectives on coherence

In most cases, these sectoral concerns may be resolved with power, such as when the dominance of some sectoral aims come at the expense of most others. Although countries focus on ‘sustainable development’, economic growth overshadows other domestic aims (Smith et al., 2014; Coffey, 2013; Battams & Townsend, 2019). Further, national security and stability trumps international aims such as peacebuilding and foreign sustainable development (Olsen, 2013: 1839; Baranyi, 2014; de Coning & Friis, 2011; Schmitz & Eimer, 2020; see also Scott & Thurston, 2004 on gendered and racialised issues in PI). In such cases, assessing a dominant discourse according to its consistency or integration – without questioning its fundamental intended impact - may miss the point.

This dominance is a focal point for critical scholarship questioning the appropriateness of the sustainable development ideal, such as to describe it as a means to reduce climate change policy ambitions while prioritising economic growth and maintaining the primacy of capitalism, rather than seek a radically new model of social, economic, and political activity (e.g. Kurze & Lenschow, 2018; Cairney et al., 2023). In that context, the main question is not ‘will this policy be sustainable?’ but rather ‘will it help or hinder growth?’. In that context, for example, sustainable development becomes a vehicle for economic issues in environmental policies (Smith et al., 2014), there is support for renewable energy if it does not undermine the use of fossil fuels as part of ‘energy security’ (Al-Sarihi & Mason, 2020: 1226; Warren et al., 2016: 8–9; Rietig, 2019: 2), transport policies may focus more on reducing emissions from cars than car use (Kivimaa and Virkamak, 2014), and climate adaptation is more politically feasible than mitigation (Rauken et al., 2015). Similarly, public health aims are mainstreamed in all policies if they do not undermine economic growth or international trade (Lencucha & Thow, 2020; Wu, 2010).

### Competing dynamics on coherence

These unresolved tensions regarding the best means to seek coherence, coupled with periods of renewed activity, may contribute to multiple waves of coherence and incoherence. Some drivers relate to *competing ways to make policy*. First, while informal subsystem activity may be the norm, it is disrupted periodically by new formalised reforms (Rouillard et al., 2013). Second, there are continuous tensions between centralised and more participatory forms of governance, such as when Mullally et al. (2018: 75) find ‘paternalist’ versus ‘deliberative’ discourses on citizen engagement in energy policy transitions.

Other drivers relate to *policy aims and priorities*. First, one dominant or overarching frame may replace another and have ripple effects across many sectors (e.g. Hommels et al., 2013: 455-6 on the impact of changing EU internal market frames). Second, moves to update agendas to raise new concerns may unearth new tensions between aims. For example, policy actors realise that climate PI is not always consistent with environmental PI, such as if using nuclear or hydro power to reduce greenhouse gas emissions, with spillovers on aims regarding biodiversity (Rietig, 2013). These problems may be amplified by the number of issues, sectors, and levels of government involved in complex PI, such as when connecting CPI to mitigation and adaptation plans, horizontally and vertically in one political system, when delivering agreements between aid-providing and aid-receiving countries, and designing land use policies for sustainability and resilience (di Gregorio et al., 2017; Göpfert et al., 2019). Third, new combinations of aims and ideas produce different expectations for the state, such as when the UK government asserts central control over public sector bodies but seeks to minimise state intervention in the market when fostering sustainable energy (Eadson, 2016). The result is a mix of different tools that are superficially coherent but are driven by different rationales, such as revenue raising fuel duties, grants and incentives for renewables, and the weak promotion of local agreements for renewable or community development projects (2016: 1620).

## Concluding discussion: there is no single policy coherence ideal to which we can all aspire

Perfect policy coherence is a rather vague and unattainable concept. The ideal-type helps to explain a coherence gap akin to a more amplified version of the classic implementation gap. Table 1’s list of requirements for perfect coherence helps to explain systematically why it is unattainable. Policymaking does not represent a simple, well-understood, and predictable process to which all actors can contribute. Policy aims receive insufficient attention or priority. Governments use the whole-of-government rhetoric without substantive analysis, engagement with trade-offs, or a clear theory of change. Cross-sectoral ideas are not a good fit with specialist subsystem activity. Collaboration is limited and superficial modes of stakeholder and citizen engagement are pervasive. High-level agreements are generally vague and non-confrontational, while some are substantive but provoke unresolved conflict. There is insufficient attention to the complexity of the policy mix, or the procedural tools required to coordinate. Such dynamics ensure that the strategy does not work as intended if implemented. The evaluation of such outcomes is lacking and measures for success are contested. Therefore, such PI strategies lack credibility and durability.

Further, empirical studies and policy theory insights identify a combination of complex policymaking dynamics and competing perspectives on coherence, to throw up the possibility of periods of coherence and incoherence over time. Most PI agendas are in ‘permanent political tension’ not only with ‘the sectoral logic of policymaking’ (Cejudo & Trein, 2023: 9) but also the dominance of ill-resourced top-down approaches and the economic and security policy aims that undermine the others. New top-down initiatives interact with the usual ways of doing things among subsystems in national and subnational government, usually without the resources or direction to make a lasting difference. A temporary burst of attention to non-dominant aims challenges the dominance of – largely economic and security – aims, usually without a substantive impact on routine practices and outcomes.

In such cases, the dynamic between drivers and barriers to PI are never really resolved. Rather, they contribute to continuous processes of agenda setting to produce modest and vague aims, or a more substantive challenge backed by limited resources. Further, the weight of dissatisfaction with progress, driven by calls for new ways of making policy and for new policy priorities, drives frequent calls for change despite limited evidence of the impact of such moves.

Finally, Table 1 suggests that the ideal-type contains potentially incoherent elements that require proper attention, such as if they combine a top-town and centralised idea of coherence, built on strategy development and implementation, with bottom-up ideas regarding decentralisation, to ensure meaningful levels of local autonomy to foster stakeholder and citizen engagement to tailor policy to local contexts. As such, the ideal-type is not only unattainable but also does not provide a clear and consistent ideal to which to aspire.

This analysis serves as a cautionary tale for subsequent waves of support for policy integration. Most published research describes vague aspiration without engaging with real world dilemmas. Most governments express vague commitment to a whole-of-government approach without engaging with issues of politics and complexity, thus delaying conflict and choices between trade-offs, and producing inconsistent ways to make and deliver policy. Hence, most research and policy contributes to a cycle of renewed then thwarted agendas for policy coherence. In that context, I show that the pursuit of ‘perfect policy coherence’ may seem like a heroic quest but is really a fool’s errand.

## Data Availability

No datasets were generated or analysed during the current study.
